# Global Pyrogeography: the Current and Future Distribution of Wildfire

**DOI:** 10.1371/journal.pone.0005102

**Published:** 2009-04-08

**Authors:** Meg A. Krawchuk, Max A. Moritz, Marc-André Parisien, Jeff Van Dorn, Katharine Hayhoe

**Affiliations:** 1 Department of Environmental Science, Policy and Management, University of California, Berkeley, California, United States of America; 2 Natural Resources Canada, Canadian Forest Service, Edmonton, Alberta, Canada; 3 ATMOS Research and Consulting, Lubbock, Texas, United States of America; 4 Department of Geosciences, Texas Tech University, Lubbock, Texas, United States of America; Centre National de la Recherche Scientifique, France

## Abstract

Climate change is expected to alter the geographic distribution of wildfire, a complex abiotic process that responds to a variety of spatial and environmental gradients. How future climate change may alter global wildfire activity, however, is still largely unknown. As a first step to quantifying potential change in global wildfire, we present a multivariate quantification of environmental drivers for the observed, current distribution of vegetation fires using statistical models of the relationship between fire activity and resources to burn, climate conditions, human influence, and lightning flash rates at a coarse spatiotemporal resolution (100 km, over one decade). We then demonstrate how these statistical models can be used to project future changes in global fire patterns, highlighting regional hotspots of change in fire probabilities under future climate conditions as simulated by a global climate model. Based on current conditions, our results illustrate how the availability of resources to burn and climate conditions conducive to combustion jointly determine why some parts of the world are fire-prone and others are fire-free. In contrast to any expectation that global warming should necessarily result in more fire, we find that regional increases in fire probabilities may be counter-balanced by decreases at other locations, due to the interplay of temperature and precipitation variables. Despite this net balance, our models predict substantial invasion and retreat of fire across large portions of the globe. These changes could have important effects on terrestrial ecosystems since alteration in fire activity may occur quite rapidly, generating ever more complex environmental challenges for species dispersing and adjusting to new climate conditions. Our findings highlight the potential for widespread impacts of climate change on wildfire, suggesting severely altered fire regimes and the need for more explicit inclusion of fire in research on global vegetation-climate change dynamics and conservation planning.

## Introduction

Wildfire is an ecological disturbance process that has a heterogeneous global distribution controlled by the coincidence of three basic requirements: vegetative resources to burn, environmental conditions that promote combustion, and ignitions. While the physical process of combustion is theoretically simple, understanding the relative influence of biotic and abiotic controls on observed, modern fire regimes is an ongoing focus in ecological research, nuanced by the role of humans who are changing landscapes to be more or less flammable, as well as lighting and extinguishing fires [1]–[3]. Interest in fire research has become global and interdisciplinary due to influences, interactions, and feedbacks among fire, terrestrial, and atmospheric systems in the context of human health [4], climate dynamics [5], and policy adaptation [6].

Recent work has begun to synthesize common trends in environmental influence on fire across broadly different locations [7], [8], but our comprehension of overarching biophysical controls on global fire activity is still limited. The collection of fire data by remote sensing provides an archive from which to examine global patterns of wildfire, such as differences between areas of the planet where fire occurs and those where it does not. The first cohort of global fire studies focused on validation and translation of remotely sensed fire products to area burned [9], [10], global carbon emissions from fire [5], and how seasonal variation in fire relates to ocean-atmosphere cycles [11]–[13]. An initial characterization of the global fire environment by Dwyer et al. [14] consisted of a short-term assessment of 21 months of data to evaluate simple relationships between fire activity and climate variables, as well as fire and vegetation type. The refinement of global fire databases and accumulation of longer-term records has further enabled such statistically-based analyses of empirical data, including relationships of global fire activity to anthropogenic explanatory variables [2] and circum-tropical fires to moisture and energy metrics [8]. However, a thorough multivariate statistical assessment that captures the complexity of broad global fire-environment relationships has yet to be undertaken. Furthermore, once macro-scale fire-environment relationships have been established, the information provided by statistical parameter estimates can be used to consider crucial questions about how climate change may alter the distribution of fire across the globe.

As an alternative to statistical models, simulations using dynamic global vegetation models (DGVMs) have been used as a complementary method to study the global distribution and effect of fire [15]–[18]. Although novel ideas about the role of fire in shaping global vegetation patterns [19] and how fire frequency might change in the future [15] have been explored with DGVMs, the simplified approach to simulating fire in many DGVMs has, as yet, limited their utility in understanding current patterns of fire around the world. As such, current DGVMs are not capable of explicitly simulating the extent to which future climate change may alter fire dynamics [20], [21], though refinements to DGVM fire modules and simulation experiments are promising.

The concept of global pyrogeography — the study of the spatial distribution of fire across the planet — borrows heavily from ecology, where three general factors are used to explain the distribution and abundance of organisms: resource availability, physiologically appropriate environmental conditions, and dispersal ability [22]. In the context of fire, flammable vegetation is the consumable resource, fire-conducive weather patterns and their long-term representation (i.e., climate) form the environmental conditions axis, and ignitions are analogous to dispersal [23]. Admittedly, these dominant constraints on the distribution of fire are intertwined and complex. Climate is a superordinate control over both the resources and conditions for fire [7], because it has a direct, short-term effect on fire weather conditions and an indirect, longer-term effect in determining the distribution and quantity of flammable vegetation to burn. In turn, weather and vegetation conditions affect ignitions, in conjunction with topographic effects on patterns of lightning strikes [24] and anthropogenic control over ignition.

**Figure 1:**
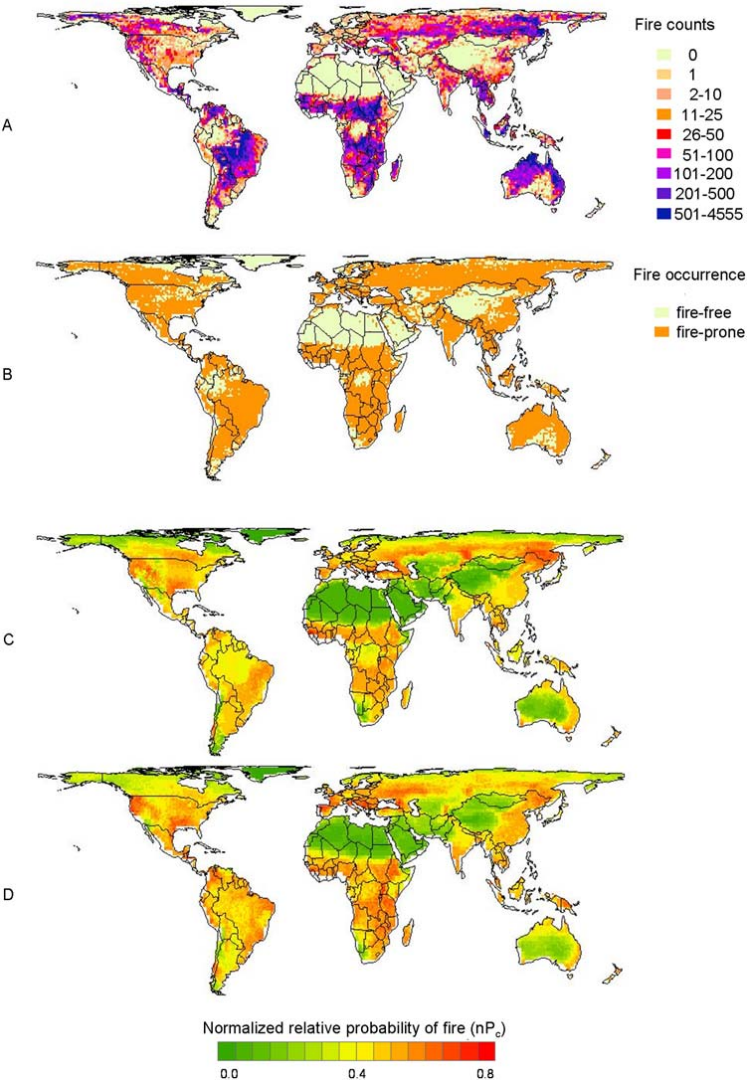
The observed and modeled distribution of fire under current conditions. (A) Cumulative counts of fire activity detected by the Along Track Scanning Radiometer (ATSR) around the world at a resolution of 100 km over 10 years. (B) The same fire data classified to represent fire-prone (orange) and fire-free (yellow) parts of the world; note that areas of white within terrestrial boundaries were clipped from the analyses to match climate data. (C) Mean of normalized relative probability of fire (nP_c_) for ten FIRE_NPP_ sub-models of fire-prone parts of the world under current conditions. (D) Mean of normalized relative probability of fire (nP_c_) for ten FIRE_noNPP_ sub-models of fire-prone parts of the world under current conditions.

Global studies examining how the distribution of fire might change in the future are necessary to establish the potential impacts of climate change on vegetation and ecosystems. Local and regional studies have projected both increases and decreases in future fire activity, [25]–[27] however we lack the quantitative estimates needed to understand what the net effect might be across a warmer planet. For these reasons, our first goal in this global pyrogeography was to characterize the observed global fire occurrence pattern (Figure 1) with an ensemble of multivariate statistical generalized additive models (GAMs) combining existing fire occurrence, climate, net primary productivity (NPP), and ignition data. Since many parts of the globe are fire-free because they have little or no vegetation to burn, it is informative to distinguish between areas that do not burn due to limiting consumable resources versus limiting environmental conditions. To address this issue, we included global vegetation distribution as an explicit metric for resource availability in one ensemble of models (FIRE_NPP_), allowing climate to describe additional variability in fire-conducive conditions. We contrasted this approach to another ensemble of models (FIRE_noNPP_) where climate variables alone were used to describe both resources and conditions needed for fire. Our results provide a novel multivariate framework to describe where we currently see wildfire across the planet. We then apply these models to future climate scenarios, providing a first estimate of potential changes in the global distribution of fire. The climate change projections presented here are based on simulations from the Geophysical Fluid Dynamics Laboratory Climate Model 2.1 (GFDL CM2.1). Our intent is to demonstrate the scope of changes that could occur given anticipated climates under mid-high (A2), and lower (B1) emissions scenarios proposed by the IPCC Special Report on Emission Scenarios [28].

## Materials and Methods

### Data

#### Overview

We constructed statistical GAMs for two regression model scenarios to characterize current fire patterns, FIRE_NPP_ (explicitly including biomass to burn) and FIRE_noNPP_ (allowing climate to explain both biomass and environmental conditions), based on ten random sub-samples of fire [29], climate [30], [31], NPP [32], and ignition [33], [34] data. We refer to these models as each forming a ‘sub-model ensemble’. The ensemble GAMs were then used with simulated future climate data to project the potential distribution of fire in the 21^st^ century. We used global data at a spatial resolution of 100-km (10 000 km^2^) on a Behrmann Cylindrical Equal Area projection, resulting in 12 098 pixels over the terrestrial extent of the planet. The Antarctic continent and small islands were excluded as were some coastal regions, because of an *a priori* cutoff rule of at least 1/3 land fraction in the gridded 2-degree climate data.

#### Fire

Mapped global vegetation fire locations came from the European Space Agency's Advanced and Along Track Scanning Radiometer (ATSR) World Fire Atlas (algorithm 2) for 1996 to 2006 [29]. The ATSR fire data were registered to our study domain, where pixels containing at least one fire over the decade of record were categorized as ‘fire-prone’ and those that did not as ‘fire-free’ (Figure 1A and 1B); alternative classifications will be explored in future work. Using this criterion, we identified 8399 (69%) pixels as fire-prone over the 10-year period. The ATSR satellite data include both human- and lightning-caused fires, which are currently indistinguishable.

There are numerous satellite sensors that can be used to record wildfire data, and these have been shown to vary somewhat in their estimates of activity and distribution of fire [35]. We selected the ATSR data because it provides the longest temporal data set (10 years) of all fine-resolution global fire products, and post-processing by Mota et al. [29] provided detailed screening of non-vegetation fires. Mota et al. [29] used volcanic activity, night-light, and land cover data as screening tools to remove non-vegetation fires from this ATSR database alongside statistical techniques that detected anomalous data clusters. The ATSR senses active night-time fires at a three day interval to a minimum burning area of 0.01 to 0.1 ha. Night-time acquisition minimizes false positives due to sun-glint, reflection, and bright soil surfaces, but it potentially misses short-duration daytime events and summer fires at high latitudes [10]. For example, Kasischke et al. [10] demonstrated that many fires may go undetected by the ATSR in the boreal forest.

The macro-scaled resolution of our study is one way to address limitations of the fire data, namely omission errors due to detection difficulties, and a relatively short temporal extent. We tested the assumption that ATSR fire data were representative of other global fire products and not biased due to detection difficulties by comparing the distribution of ATSR fire data to those produced by the newly available MODIS Collection 5 active fire data [36], and found the distribution to be very similar (Text S1, Figure S1). We also tested whether the decade of ATSR fire data were representative and spatially similar to long-term fire patterns by comparing ATSR data to a map of large forest fires recorded in Canada between 1959 to 2002 [37] (Text S2, Figure S2). We found a strong accord between our macro-scaled ATSR product and the Canadian fire database, even in the northern boreal forests where detection of fire can be compromised in finer-scaled studies [10].

#### Climate

Our statistical GAMs were built from 17 climate variables (Table 1) representing potential environmental conditions controlling fire. These so-called ‘bioclimatic variables’ were calculated climate averages of temperature and precipitation [30], providing biologically meaningful approximations of recent historical energy and water balances, as well as environmental extremes. We used variables calculated for climate data from GFDL CM2.1 [31] General Circulation Model (GCM) historical simulations (1961 to 1990) and from observed climate normals (1950 to 2000) provided by WorldClim to generate statistical estimates from GAMs (see *Regression modeling*, below and Text S3, Figure S3). The GFDL CM2.1 is a global coupled climate model developed at NOAA's Geophysical Fluid Dynamics Laboratory and was designed to simulate oceanic and atmospheric climate and variability over a multi-century temporal extent, at a diurnal resolution [31]. WorldClim is a dataset of interpolated climate surfaces generated using thin-plate smoothing splines from weather station data recorded around the world [30]. The GFDL CM2.1 and WorldClim-based models had very similar shapes and effect sizes, so given this consistency, we built our wildfire occurrence models from the GFDL CM2.1 historical simulation data, from which future fire could then be projected seamlessly using global 30-year climate averages of variables simulated for time periods 2010–2039, 2040–2069, and 2070–2099. The three time periods of simulated future data used in our study represent future climate conditions corresponding to increasing concentrations of CO_2_.

**Table 1:** Environmental variables used in regression analyses.

Variable	Description and Units
**Climate**	Derived from monthly temperature and rainfall values
Annual mean temperature	°C
Mean diurnal range	mean of monthly (max temp−min temp), °C
Isothermality	mean diurnal range/temperature annual range (×100)
Temperature seasonality	standard deviation of temperature (×100)
Maximum temperature of warmest month	°C
Minimum temperature of coldest month	°C
Temperature annual range	maximum temperature of warmest month – minimum temperature of coldest month, °C
Mean temperature of wettest month	°C
Mean temperature of driest month	°C
Mean temperature of warmest month	°C
Mean temperature of coldest month	°C
Annual precipitation	mm/year
Precipitation of wettest month	mm/day
Precipitation of driest month	mm/day
Precipitation seasonality	coefficient of variation
Precipitation of warmest month	mm/day
Precipitation of coldest month	mm/day
**Vegetation**	
Net primary productivity (NPP)	amount of solar energy converted to plant organic matter through photosynthesis (g C per 0.25 decimal degree cell/year).
**Ignitions**	
Lightning flash density	flashes/km^2^/day
Human footprint	normalized gradient of human influence (0 to 100)

The GFDL CM2.1 simulations for historical and future climate conditions were generated with a resolution of 2 degrees [31] and re-gridded to a 100-km resolution across the globe in order to provide a standardized format compatible with the resolution of this study (i.e., no statistical downscaling was performed). Historical model simulations (pre-2000) corresponded to the Coupled Model Inter-comparison Project “Twentieth Century Climate in Coupled Models” or 20C3M scenarios [38] which represent the best efforts to reproduce observed climate over the past century. Future GFDL CM2.1 simulations (2010–2100) used here for fire-climate change projections were forced by the IPCC Special Report on Emission Scenarios (SRES, [28]) mid-high (A2) emission scenario, in which CO_2_ concentrations reach 830 ppm by 2100. A lower emissions scenario, B1, which can be viewed as a proxy for stabilizing atmospheric CO_2_ concentrations at or above 550 ppm by 2100, was also examined for comparison. Because of the known variability in and among GCM outcomes, we also compared the GFDL CM2.1 future projections of the most significant climate variables identified in the regression models with simulations from 15 other atmosphere-ocean general circulation models (AOGCMs) archived by the IPCC Fourth Assessment Report Working Group 1 Program for Climate Model Diagnostics and Intercomparison (PCMDI) database as a simple assessment of uncertainty in the GFDL-based fire projections.

#### Vegetation

We quantified the broad-scaled distribution of flammable vegetation using NPP (Figure S4). Measures of NPP represent the amount of solar energy converted to plant organic matter through photosynthesis quantified as elemental units of carbon per unit time and area, whereas vegetation to burn is ostensibly the standing stock of biomass represented as units of carbon per area. The approximately linear relationship between NPP and biomass [39] invites the use of NPP as a metric of flammable vegetation, since detailed spatially-gridded, globally extensive measures of biomass are not readily available [39]. Mapped global NPP was provided by the Carnegie-Ames-Stanford Approach (CASA) terrestrial carbon model [40] at a resolution of 0.25 degrees. Estimates of NPP can vary according to the data and method used; here we used estimates created by Imhoff and Bounoua [32] using climatology, land cover, solar radiation, soil texture and vegetation data (AVHRR from 1982–1998) described therein. We aggregated the raw values to our 100-km sampling grid using the maximum NPP value recorded from each pixel. Areas of persistent snow cover (136 pixels) for which no NPP data were available were given a value of zero.

#### Ignitions

We examined the potential for human ignition to limit fire distribution using the Human Footprint (HF) dataset from the Last of the Wild Project [33] as a proxy for ignition potential. The HF describes human population pressure, land use and infrastructure, and access. Lightning, the other major cause of ignitions, was assessed using the NASA Global Hydrology and Climate Centre Lightning Team's high resolution annual lightning climatology which reports annual flash rates per km^2^ from data collected between 1995 and 2005 [34]. The results of a supplemental analysis indicated that there were few areas of the planet where ignition may be a limiting factor for the 10-year 100-km resolution of our study (Text S4). Of note, the ignition indices we used did not distinguish specific lightning characteristics or human behaviors required for fire ignition. Although ignition potential was almost never limiting, we still included ignition agents in the regression models to test whether they reflected any variation in the likelihood of fire occurring.

### Regression modeling

To estimate environmental controls of fire occurrence, we chose a used-versus-available sampling design analogous to resource selection functions used in studies of wildlife distribution [41]. This design allowed us to quantify the particular resources and conditions conducive to fire by contrasting pixels where fires occurred against a random sample of pixels using statistical models that estimate a relative probability of occurrence. We did not follow a used-versus-unused design because our comparison between ATSR fire data and the Canadian large fire database (Text S2, Figure S2) demonstrated that despite the overall similarity between the databases, fires detected by ATSR during the 1996–2006 time period do not represent *all* pixels that are fire-prone. We used GAMs [42] for statistical modeling in R [43] to provide flexibility in describing nonlinear relationships between fire occurrence and environmental variables.

For the FIRE_NPP_ (explicitly including biomass to burn) and FIRE_noNPP_ (allowing climate to explain both biomass and environmental conditions) scenarios, our sub-model ensemble approach limited spatial structure in the data, included among-sample variability, and allowed model cross-validation. Addressing spatial dependence was particularly important since spatial data require careful consideration in statistics due to the effect of autocorrelation on variable [44], [45] and model [46] selection. Data for each sub-model were selected by taking a 15% random sample (n = 1 260) with replacement from the ‘used’ data (pixels where fire was detected) and the equivalent number of samples from the ‘available’ data (all pixels). We chose the 15% sample fraction since variograms of response (fire) and predictor (climate and NPP) variables indicated the beginnings of a sill in semivariance at a distance of 15 to 22 pixels (≈2 000 km).

We used the GAMs to identify simple and interpretable forms of candidate variables that described the distribution of fire-prone parts of the world. Our goal was to develop models that explained strong patterns of variation in fire distribution while not over-fitting the observed data. In keeping with this goal, we used the Akaike Information Criterion (AIC) as a model selection tool because it is based on the principle of parsimony [47].

Multiple phases were required for model selection and development. In the FIRE_NPP_ models, we first estimated the relationship between fire occurrence and NPP (Figure S4) to account for variation in resources to burn, and held this relationship constant for subsequent model development by using an offset term. In each of the ten sub-models, the AIC indicated that the most parsimonious form of the NPP offset term was estimated with three degrees of freedom, a measure of complexity in the shape of the relationship. After the inclusion of the NPP offset, model development proceeded identically for variable selection in FIRE_NPP_ and FIRE_noNPP_ scenarios. Each sub-model of the ensemble was developed using a forward selection procedure. Variables were included in an order decided *a priori* by rank according to the AIC estimated on independent relationships between fire occurrence and each environmental variable. The most parsimonious form of the variable was subsequently selected using AIC and visual assessment of plots showing the main effect, standard error estimates, distribution of the data, and residuals. A reduction in AIC of more than six was required for the inclusion of the variable in a sub-model.

Explanatory variables strongly correlated to one another were flagged *a priori* based on scatter plots and Pearson correlation coefficients. Although multicollinearity does not affect the use of the model to infer the mean response under observed conditions, it can make interpretation of variables difficult because parameter estimates are conditional on other variables in the model, and valid predictions can only be made if multicollinearity patterns hold for the new data [48]. Therefore, as additional terms were added to each GAM, we checked for changes in the shape and explanatory power of the existing variables. Variables that entered the model earliest took priority; lower-ranked variables were omitted if they were strongly collinear and altered the existing relationships, even if they otherwise reduced AIC sufficiently.

We assessed the predictive performance of each of the FIRE_NPP_ and FIRE_noNPP_ sub-models using a random sub-sample cross validation method [49]. Cross validation compares model predictions of training data against a withheld set of data, and the method proposed by Johnson et al. [49] is the most appropriate for a used-versus-available sampling design: tests are done on the correlation between binned estimated values of relative probability from each model and the frequency of independent withheld values (observed) in the same bin (here, 30 bins, each with a width of 0.1). The two most important metrics of those proposed by Johnson et al. [49] were the tests indicating: i) whether the model is better than random as indicated by a slope of the regression line between the observed and estimated values significantly different from zero; and ii) whether the model fits the data well as indicated by the R^2^ value of the relationship between these observed and estimated values.

We ranked the overall importance of explanatory variables from the ten FIRE_NPP_ and FIRE_noNPP_ sub-models by summarizing the number of times they were selected in the ensemble, as well as the mean change in AIC when each was removed from a given sub-model. We also plotted the shape of the dependent response to each variable to identify and interpret the dominant form of each relationship.

To illustrate the spatial distribution of fire under current climate conditions, we calculated a normalized index of relative probability scaled between zero and one from parameter estimates of the sub-model ensembles. Calculations excluded the intercept because it is not informative in the used-versus-available study design [50]. First, the relative probability for current conditions (rP_c_) for each sub-model was calculated as:
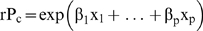
(1)where β_p_ are the parameter estimates for each environmental variable, x_p_. We then normalized these relative probabilities for each sub-model and took the mean of the ensemble. The normalized relative probability for current conditions (nP_c_) for each sub-model was calculated as:
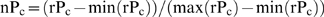
(2)


### Projection of global fire distribution under future climate conditions

Parameter estimates from the GAMs were applied to future climate simulations to generate projections of future fire distribution. Future climate conditions were estimated for the time periods 2010–2039, 2040–2069, and 2070–2099 using the SRES A2 and B1 emissions scenarios. Several methods are available to generate climate change projections from AOGCM data [51], for example, by using output directly generated by AOGCMs or by adding an anomaly or delta value, calculated as the difference between future and present conditions as simulated by an AOGCM, to observations. We examined these two approaches and found that, because of the consistency between fire-climate relationships estimated for observed and simulated current climate conditions (Text S3, Figure S3), the first of these approaches would provide equivalent information to the second, while retaining the spatial correlation inherent to the physical model that generated the simulations.

The two GAM ensembles present different ways to think about the future of fire. The FIRE_NPP_ model depicts what the change in fire distribution might be if the future global pattern of NPP remained constant; we did not generate climate change projections from the CASA productivity models, so NPP was essentially held constant in our FIRE_NPP_ model projections. This scenario is obviously unrealistic over the longer term because of the strong links between climate and vegetation, but for near-future projections such as 2010–2039, it may be reasonable to presume a relatively constant NPP, given that climate induced changes in fire are expected to occur more quickly than substantial changes in vegetation via range shifts [52], [53]. In contrast, the FIRE_noNPP_ models predict what the future distribution of fire might be under the assumption that the climate variables in the regression models jointly describe vegetation patterns (productivity and structural form) as well as fire weather conditions. These predictions may provide an overly liberal view of the near future, because they essentially remove the dispersal constraints of vegetation change. However, projections from these FIRE_noNPP_ ensembles could be more representative of what might be expected later in the century, such as 2070–2099.

We used a delta index (P_Δ_) to assess the differences in current and future fire distributions. For the P_Δ_ index, we first calculated the normalized relative probability of fire for the future (nP_f_) using Equation 1, but based on future climate conditions. We then quantified the changes between future and current relative probability of fire for each sub-model as:
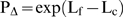
(3)where L_f_ = ln(rP_f_) and L_c_ = ln(rP_c_) and L_f_ and L_c_ are relative probabilities of the current and future models, respectively. A P_Δ_ of less than one indicates a reduction in fire, whereas a value greater than one indicates an increase. We calculated three delta indices, P_Δ1039_, P_Δ4069_, and P_Δ7099_, for time periods 2010–2039, 2040–2069, and 2070–2099, restricting the ranges of climate values for future projections to those of the training models to avoid spurious prediction. Since analogues existed for virtually all future climate values, this restriction did not overly constrain projections. We also removed the terms estimating the relationship between fire occurrence and lightning flashes from the sub-models where it was selected, as no information was available to estimate future lightning patterns from AOGCM simulations.

Lastly, we identified potential “hotspots of change” where fire was projected to i) invade, by increasing in locations where current probabilities of fire were low; and ii) retreat, by decreasing in locations where current probabilities of fire were high. To highlight the spatial extent and specific locations with the most potential for near-term shifts, we mapped the distributions of fire invasion and retreat for scenario A2 at time period 2010–2039 from the FIRE_NPP_ ensembles (i.e., P_Δ1039_), masking out regions of the globe with NPP currently less than 96 gC/m^2^/year (e.g., Arctic, Sahara, Greenland). Although this excludes ∼21% of terrestrial lands that now lack biomass to burn, it also underestimates the amount of future fire invasion into areas where vegetation could begin to establish in the next few decades. Selection of the nP_c_ threshold values to isolate areas with relatively low (for invasion) and high (for retreat) current probabilities of fire was based on the distribution in values of modeled fire probabilities around the median value of the current FIRE_NPP_ ensemble.

## Results

### Statistical modeling of present-day influences on fire distribution

Statistical modeling using the GAMs indicates that both resources and conditions contribute to discriminating fire-prone parts of the world, with similar relationships in both FIRE_NPP_ and FIRE_noNPP_ model ensembles (Table 2, Text S3, Figure S3). Vegetation NPP had the strongest single relationship of any predictor variable to the distribution of fire (Table 2, Figure S4), and eleven additional predictors were selected in both FIRE_NPP_ (after accounting for NPP) and FIRE_noNPP_ ensembles (Table 2). The maximum number of predictors included in a single sub-model was seven, and this occurred only once; the mode was five. Estimated degrees of freedom for the majority of variables ranged between one and five, generally resulting in simple response curves. There were limited differences between the predictors selected in the FIRE_NPP_ and FIRE_noNPP_ ensembles (Table 2) and between the spatial distributions of expected fire probabilities (Figures 1C and 1D). For example, temperature seasonality was only selected in FIRE_NPP_ models but a closely allied variable, temperature annual range, was selected in both FIRE_NPP_ and FIRE_noNPP_ models (Table 2). The human footprint (HF) metric and lightning flash density explained some variability in fire occurrence, but only when NPP was not included in the model (Table 2).

**Table 2:** The ranked importance of variables selected in FIRE_NPP_ and FIRE_noNPP_ sub-models based on the number of times the explanatory variable was selected (SEL) and the mean change in AIC value, which was used to measure the relative amount of variation explained.

Variable	FIRE_NPP_		FIRE_noNPP_	
	SEL*	AIC*	SEL	AIC
Net primary productivity	10	125	*na*	*na*
Mean temperature of warmest month	9	16	10	30
Annual precipitation	7	14	10	79
Mean temperature of wettest month	5	13	4	10
Temperature seasonality/temperature annual range	3/2^#^	14/25	0/3	*na*/12
Mean diurnal range	3	10	4	15
Precipitation of driest month	3	7	3	12
Lightning flash density	2	13	5	10
Mean temperature of driest month	2	7	3	12
Precipitation of coldest month	1	13	0	*na*
Human footprint (HF)	1	10	6	12

* Explanatory variables separated by ‘/’ are highly correlated and were never selected together in a model, but represented similar environmental trends in current conditions.

Model cross-validation indicated good discrimination of fire-prone parts of the world, with tests showing sub-models of both the FIRE_NPP_ and FIRE_noNPP_ ensembles to be significantly better than random. This diagnostic was demonstrated by slopes of the correlations between estimated and observed values of relative probability that were all significantly different from zero, and R^2^ values between estimated and observed data ranging between 0.96 to 0.98 for the FIRE_NPP_ model ensemble and 0.94 to 0.98 for FIRE_noNPP_ ensembles. Visually, the FIRE_NPP_ model ensemble provided finer discrimination of fire-prone parts of the world (Figures 1C and 1D), especially in regions where resource levels are high such as the tropics, illustrating areas where climate variables (FIRE_noNPP_) were less able than NPP (FIRE_NPP_) to capture variation in fire occurrence. For example, the FIRE_noNPP_ ensemble predicted little variation in the probability of fire across the Amazon and Congo regions of South America and Africa (Figure 1D) despite containing large contiguous patches of fire-free areas at the centre of these regions in the observed data (Figure 1B).

### Projection of global fire distribution under future climate conditions

**Figure 2:**
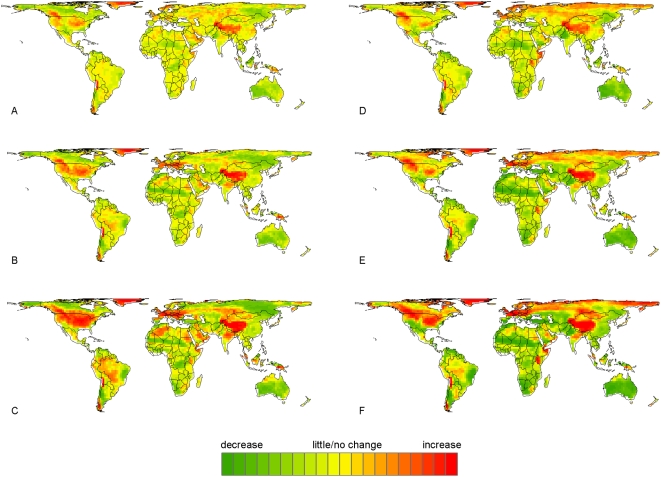
Changes in the global distribution of fire-prone pixels under the A2 (mid-high) emissions scenario. An increase from current conditions (red) is indicated by a P_Δ_ greater than unity, little or no change (yellow) is indicated by a P_Δ_ around unit, and a decrease (green) is indicated by a P_Δ_ less than unity. Panels show the mean P_Δ_ for the ensemble of ten FIRE_NPP_ (A–C) and FIRE_noNPP_ (D–F) sub-models. Climate projections include 2010–2039 (A, D), 2040–2069 (B, E) and 2070–2099 (C, F).

Given the success of the statistical models in reproducing present-day fire distributions, we then applied the models to estimate the change in future fire probabilities (P_Δ_) resulting from the A2 (mid-high emissions) and B1 (lower emissions) climate projections generated by the GFDL CM2.1 AOGCM. Projected decreases in fire were indicated by values less than 1.0 and increases by values greater than 1.0 (Figure 2 and Figures S5, S6). For the A2 scenario, projected changes in fire over all time periods ranged from 0.5 to 2.8, depending on the statistical sub-model used and the geographic location; corresponding results for B1 ranged from 0.7 to 1.9 (Figure 2 and Figures S5, S6). Despite changes in fire probabilities that deviated progressively more from current conditions over time and with a higher emissions scenario, Figure S6 illustrates roughly equivalent increases and decreases in fire probability over the globe. The coarse spatio-temporal scale used for this study allowed projections of change without including finer scaled details known to affect local fire activity such as time since last fire, since the likelihood of a fire burning through all biomass available in each 10 000 km^2^ pixel is relatively unlikely.

It is important to note that the projections shown here are based on simulations from one AOGCM only. This was a deliberate choice, as our primary purpose was to describe the development of the statistical modeling technique and explore its potential application to future projections of wildfire. Some measure of the robustness of these projections can nonetheless be obtained through comparison of GFDL CM2.1 projections with the average of those projected by simulations of 15 other AOGCMs archived in the PCMDI database for the three most significant climate predictors identified by the statistical analysis: mean temperature of the warmest month, annual precipitation, and mean temperature of the wettest month (Table 2). This comparison, shown in Figures S7, S8, S9, suggests that our results may in fact be indicative of the general magnitude and direction of projected changes expected from a larger number of AOGCMs. Specifically, the projections used here appear relatively conservative, close to, or below the AOGCM ensemble average for the two temperature-related variables. For precipitation, GFDL CM2.1 projections tended to lie in the lower half of the distribution, suggesting a slight tendency towards drier conditions.

Less change in P_Δ_ values occurred in FIRE_NPP_ than FIRE_noNPP_ sub-model ensembles, largely as a function of including constant vegetation patterns in the FIRE_NPP_ scenario. Both scenarios showed increasingly higher variability through 2010–2039, 2040–2069, and 2070–2099 conditions (Figure S6), which translated to fire distributions that were increasingly dissimilar to those under current conditions. In terms of geographic location, vast portions of the continental land area, particularly across North America and Eurasia, are projected to experience relatively large changes in fire probabilities (Figure 2 and Figure S5). There were obvious differences in P_Δ_ values predicted by FIRE_NPP_ and FIRE_noNPP_ models in northern regions of North America and Eurasia (Figure 2 and Figure S5), which can be attributed to the absence of the static NPP variable in the FIRE_noNPP_ model. The remaining parts of the world had relatively similar changes predicted by the FIRE_NPP_ and FIRE_noNPP_ models.

**Figure 3:**
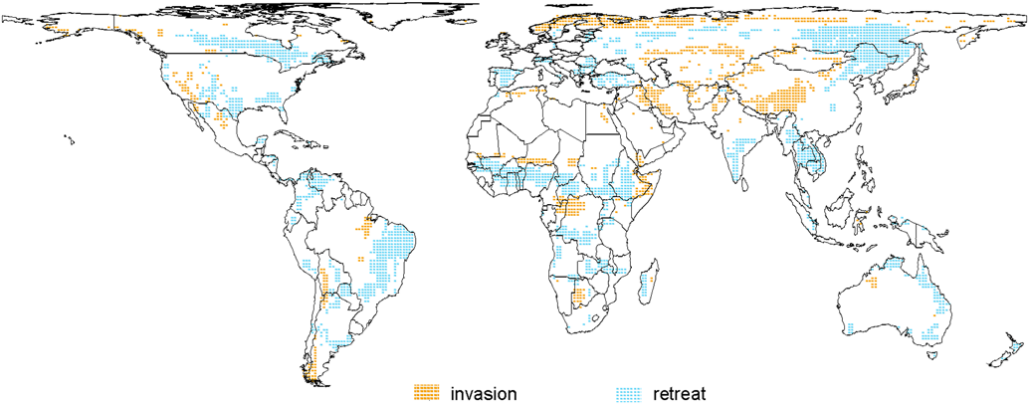
Potential invasion and retreat of fire. The invasion (orange) and retreat (blue) of fire projected by 2010–2039 under the A2 (mid-high) emissions scenario and based on the FIRE_NPP_ ensembles. Invasion was constrained to places with existing vegetation.

Areas of projected fire invasion and fire retreat for the near-term (2010–2039) given A2 emissions using the FIRE_NPP_ ensemble are shown in Figure 3. As described earlier, invasions are defined by increasing probability of fire in locations with relatively low current probabilities, and retreat by decreasing probability of fire in locations with relatively high current probabilities. Current fire probabilities (Figure 1C) exhibited a median of 0.42, and values 0.08 above and below the median were selected as cutoffs for current low and high probabilities, respectively. Of the terrestrial biosphere, 79% of lands met our conservative minimum NPP criteria, 21% were classified as currently low probability areas susceptible to fire invasion, and 38% as high probability areas susceptible to fire retreat. Although other criteria are worth considering, it appears likely that a substantial fraction of all terrestrial lands on the planet (one quarter, or 34 M km^2^ based on the climate projections used here), may be classified with invasion (∼9% of lands) or retreat (∼19% of lands) of fire (Figure 3).

## Discussion

### Global pyrogeography under current conditions

Wildfire-prone parts of the world span ecological systems ranging from tropical savannas to boreal forests, characterized by the interplay of key variables that represent resources and conditions required for fire activity. As one would expect, we found that biomass to burn is necessary for wildfires to occur: low levels of vegetative resources to burn, here represented by low NPP, resulted in a low probability of fire in areas such as desert and tundra. In our statistical models, the likelihood of fire increased with vegetation productivity. This trend has a limit, however, as other environmental factors become constraints on fire activity. For example, although some of the most biomass-rich forests of the planet, such as in peripheral Amazonia and Indonesia, can be fire-prone, the majority of closed tropical evergreen forests of the central Amazon and the Congo are relatively fire-free. These fire-free areas with high NPP rarely experience environmental conditions that promote biomass burning — seasonality, episodic wind events, low moisture levels, or ignitions — given that burnable resources are readily available. Even during anomalously dry periods, the closed canopy structure of these biomass-rich rainforests maintains a relatively high humidity that inhibits burning [54], [55]. Our analyses identified three dominant climate conditions that represent these constraints at a macro-scale: mean temperature of the warmest month, annual precipitation, and mean temperature of the wettest month.

The spatial variability in fire occurrence observed across tropical forests emphasizes the inextricable relationship between humans and fire, in that fire is dispersed by humans into areas where resources and conditions would not typically support it [1], [55]. Humans have introduced fire to biomass-rich areas either by igniting fires within fire-conducive windows of time [56] or by altering the virtually “fire-proof” vegetation structure [54], often in association with drought [57]. Although some tropical areas have been relatively fire-prone for centuries [58], [59], many areas of wet tropical rainforest which only rarely experienced fire in the past now burn due to accelerating anthropogenic pressure [60]. We found that the overall heterogeneity of fire occurrence in biomass-rich areas resulted in an asymptotic, yet somewhat decreasing, probability of burning at the highest NPP levels. The accompanying variation among our sub-models demonstrates localized differences in fire activity reflecting very high-NPP areas as ‘fire frontiers’, or areas undergoing rapid changes in human and fire activity. Both wet and wet-dry tropical forests are now at the frontier of anthropogenic development, an ever-advancing zone that has long been equated with elevated biomass burning due to land clearing by humans [61].

If the influence of humans essentially means that all areas of the world supporting sufficient biomass are potentially burnable and few are ignition-limited, how do we interpret the influence of environmental conditions on wildfire activity? Clearly, human activity has been breaking down pyrogeographic barriers [3], [60], [61] that regulate lightning-caused fire since the first use of fire by humans, but our findings indicate that environmental resources and conditions still play strong roles in determining the global distribution of vegetation wildfire. Though the spatial patterns of people, lightning, and biomass are related to some extent, resources and climate-based variables were stronger constraints on fire occurrence than ignition-related variables at the coarse resolution we used. This being said, additional change in the dynamics of fire management and/or human land use will almost certainly contribute to altering the future global distribution of fire alongside climate, presenting a wildcard in future fire-proneness.

Our finding that simple temperature and precipitation gradients consistently surface as major controls of fire supports an analogy between the broad distributions of fire-prone areas and Whittaker's [62] seminal categorization of global biomes based on these two environmental gradients. However, our study also emphasizes the potential use of synthetic variables to describe the coincident interactions of energy and water balances. Though some climate variables considered in our analysis combined elements of precipitation and temperature such as mean temperature of the wettest month, none explicitly calculated effective levels of moisture. For instance, water balance metrics [63], [64] have been shown to provide good discrimination of the occurrence, abundance, and diversity of some biota at macro-ecological scales [64]. Dwyer et al. [14] showed that at a global scale the number of months per year exhibiting a water deficit was strongly associated with observed fires. The development of global versions of existing fire-weather/climate metrics such as the Canadian Fire Weather Index [65], Nesterov Index, or novel metrics such as fire-driven deforestation potential [8], would also inform syntheses of fire patterns at broad scales.

### Global climate change and fire

Our study demonstrates a new method of examining the future of global fire activity using AOGCM-based climate projections to drive statistical models of fire activity. Initial application to simulations by the GFDL CM2.1 model under mid-high and lower anthropogenic emissions provides some striking future outcomes that encourage further development and application of this framework to more fire metrics and a broader set of AOGCM simulations. Under the future climate conditions we examined, a major redistribution of fire-prone areas occurs, with larger changes observed under scenarios of higher emissions and further into the future. Yet the net outcome implies that while parts of the world may experience regional increases in fire activity, others experience roughly equivalent decreases. Although recent perceived increases in fire through many parts of western North America are causing ecological, economic, and social concern [6], [66], our results suggest a challenge to any simplistic view that climate change will lead to more fire in all locations. Rather, we find that the interplay of changing temperature and precipitation might result in a rearrangement of global fire probabilities overall, even as global temperature increases. This does not however, imply that ecological or social impacts will be minor. Since projected changes were highly regional and our simulations suggest the potential for differences that increase across 2010–2039, 2040–2069, and 2070–2099, fire activity at a given location may become progressively altered from current conditions be it through an increase or a decrease in the likelihood of its occurrence.

Although our study illustrates the magnitude and types of changes in fire that could be expected in the near future, the quantitative findings should be interpreted with a suite of caveats. As already mentioned, additional projections based explicitly on output from multiple AOGCMs are clearly necessary. Our statistical models do not incorporate fire-climate-vegetation feedbacks that could have a further warming effect on global climate (e.g., through fire-related emissions); in this sense our projections should be seen as conservative in the amount of potential change that will occur. In addition, changes in climate will affect other natural disturbances such as insect outbreaks that kill or defoliate trees [67], and the result of interactions between these phenomena and fire activity will be very difficult to predict. Fire occurrence is only one parameter of a fire regime, and additional studies are necessary to examine potential changes in other components such as area burned, fire intensity or seasonality. Furthermore, the evolution of fire management through suppression techniques, public awareness, and policy changes is also likely to change fire activity in the future.

Projections of fire occurrence were carried out using a pair of modeling ensembles, with one scenario holding biomass structure constant at current levels (FIRE_NPP_), and one scenario where vegetation essentially tracks climate changes (FIRE_noNPP_). In the latter, larger changes were observed in fire probabilities, generated by the compensatory, larger overall effect sizes estimated for climate variables in the FIRE_noNPP_ models, including annual precipitation and mean temperature of the warmest month. Emergent differences between projections from FIRE_NPP_ and FIRE_noNPP_ ensembles were most apparent in the far north of North America and Eurasia suggesting that some environmental conditions conducive to combustion and fire spread are likely to increase there over the next decades, yet the limited availability of biomass to burn, as demonstrated by models where NPP was held constant, could buffer dramatic near-future increases in fire activity. The remainder of the world showed similar changes in the future distribution of fire for both model ensembles. We contended earlier that since the FIRE_noNPP_ ensembles for 2070–2099 represented a scenario that notionally included a shift in biomass patterns, it was more appropriate for longer-term projections due to the inevitable but slow range shifts in vegetation expected with climate change [52].

We classified areas projected to transition from low to high probabilities of fire in the near future (2010–2039) as at risk of fire invasion and areas projected to transition from high to low as at risk of fire retreat. An ecosystem that has experienced little or no fire which then incurs a higher probability or frequency of fire, such as a desert or rainforest, may be fire-sensitive and particularly susceptible to changes in community structure or ecosystem state due to increases in fire activity [60], [68], [69]. At the other end of the spectrum, decreases in fire may also affect species or communities that have adaptations that enable them to thrive in fire-prone ecosystems and may depend on narrow ranges of fire intervals for persistence. In nature, species are not simply adapted to fire, but to a given set of parameters that represent a fire regime. Our fire invasion and retreat metrics thus identify potential ‘hotspots of change’ where altered fire-proneness may catalyze relatively rapid changes in ecosystem structure, acting alongside the more gradual effect of climate on individual species tolerances. In these hotspots of change, it would be particularly valuable to quantify changes in additional fire regime parameters, for example fire intensity, a measure that includes not only changes in the conditions for fire, but also the resources available to burn under those conditions.

The rate at which fire activity may change in the future relative to rates of climate-induced changes in vegetation ranges is highly uncertain. Rapid vegetation changes are possible, such as when non-native grasses rapidly invade desert systems under suitable environmental conditions [70], [71]. However, the potential for relatively rapid and large changes in fire probabilities seen in our FIRE_NPP_ ensembles for 2010–2039 illustrate that in the near term, fire activity could change faster than many terrestrial species may be able to accommodate. For models projecting the future of species distributions, especially that of plants [72], such rapid change underlines the importance of developing methods to explicitly integrate how fire activity affects vegetation, in addition to species range changes based on plant-climate relationships alone.

Our models provide global, quantitative projections of wildfire that can be compared to existing studies of climate change to gauge not only their agreement in scope and location, but also disparities that can direct refinements in subsequent studies. For example, using a relatively simple parameterization of fire in a DGVM driven by input from multiple AOGCMs, Scholze et al. [15] describe global changes in wildfire frequency that align with our estimates in many areas, also generally supporting the concept of a net global balance between increases and decreases in future fire. In fact, such consilience between two very different modeling frameworks raises the possibility of new hypotheses about energetically-regulated limits that amount to a global “carrying capacity” for fire. We are unaware of other global studies estimating climate-induced changes in wildfire, so our statistical framework provides a new, much-needed and complementary approach to predicting future global pyrogeography.

Studies of climate-induced changes in fire have been implemented at regional scales, and these can also be used to interpret our results. For example, a DGVM and output from a GCM was used to simulate future fire regimes in Alaska, predicting a relative decrease in future area burned in central Alaska and increases in future area burned along the southern and western coasts [73], which match projections from our models. Using a regression approach similar to ours, anticipated changes in fire return interval were shown across boreal regions of North America by the end this century [26] that correspond most closely with our models where biomass was unconstrained. High-resolution regional climate simulations were used to suggest increased future fire risk across northern and eastern Australia [27], which aligns with outcomes from our models with biomass constrained, though our projections suggest more of a long-term decrease in fire in our unconstrained models. Finally, projected changes in fire weather indices for North America and Europe using simulated data from the Canadian GCM [74] are in accord with our projections in central and eastern France, but not in Fennoscandia.

### Conclusion

In this study, we first developed a statistical modeling framework capable of reproducing current-day global fire patterns and describing the influence of underlying environmental controls on those patterns. We then examined the global scope of, and potential regions likely to be affected by, severely altered probabilities of fire using statistical models and an illustrative set of climate projections. Our global pyrogeography provides a new, multivariate quantification of the current distribution of vegetation fires across the planet that is both coherent with our knowledge of global fire patterns and capable of projecting potential changes in wildfire for the future.

The original impetus for this work was to complement the subjective, expert-driven assessment of global fire regimes devised in the Global Fire Assessment, spearheaded by The Nature Conservancy [75]. Our hope is that this approach to global pyrogeography will continue to develop as a framework for providing robust estimates of potential perturbations in global fire patterns and future ecosystem changes, which could then complement and inform global DGVM simulations. Our proposed framework would also benefit from the inclusion of more advanced assessment of fire-human dynamics, the use of additional fire metrics (e.g., area burned, intensity, seasonality), updates in global fire products (e.g., MODIS), and the quantification of AOGCM-related uncertainty [76], as this information becomes available. Given the dearth of information on global fire in the context of climate change [77], the utility and importance of coarse spatiotemporal studies can only increase, providing informative and synthetic insights about global wildfire and the extent of changes that could be expected in the future.

## Supporting Information

Figure S1 The distribution of fire detected by MODIS. Data are displayed as the occurrence of fire at a spatial resolution of 100 km, between November 2000 and December 2006. Note that areas of white within terrestrial boundaries were clipped to match the fire-climate analyses.
(0.40 MB TIF)

Figure S2 Spatial comparison between a decade of ATSR fire data and fires recorded in the Canadian Large Fire Database (LFDB). Grey represents areas where no fire was detected, red shows areas where fire was detected in both the ATSR and LFDB, orange shows areas where fires were only detected by ATSR, and yellow shows areas where fires were only documented in the LFDB.
(0.09 MB TIF)

Figure S3 The modeled response, f(x), for the five most highly ranked climate variables of the FIRE_NPP_ ensemble. Response curves were estimated from fire occurrence and simulated GFDL CM2.1 data (A), and observed WorldClim data (B). Grey lines are estimates from each of the sub-models in the ensemble and black lines are the mean of these estimates. Descriptions of climate variables are found in Table 1 of the main text. Note that plotting axes vary among the variables; the x-axis for “Annual precipitation” is presented on a log10 scale.
(0.13 MB TIF)

Figure S4 The global distribution of NPP, and the relationship between fire occurrence and NPP estimated with the ten FIRE_NPP_ sub-models. Values on x-axis are presented as approximate g C/m^2^/year, by dividing data (g C/0.25 decimal degree cell) by 7.7×10^8^. The values for NPP are clipped to the extent of the GFDL CM2.1 climate data used in the regression models, such that areas of white along some coast-lines indicate areas not included in the study.
(1.09 MB TIF)

Figure S5 Changes in the global distribution of fire-prone pixels under the B1 (low) emissions scenario. An increase from current conditions (red) is indicated by P_Δ_ greater than unity, little or no change (yellow) is indicated by P_Δ_ around unity, and a decrease (green) is indicated by P_Δ_ less than unity. Panels show the mean P_Δ_ for the ensemble of ten FIRE_NPP_ (A–C) and FIRE_noNPP_ (D–F) sub-models. Climate projections include 2010–2039 (A, D), 2040–2069 (B, E) and 2070–2099 (C, F).
(1.38 MB TIF)

Figure S6 Distribution in values of change in the relative probability of fire (P_Δ_) under future conditions.
(0.55 MB TIF)

Figure S7 A comparison of mean temperature of the warmest month from 15 AOGCMs. Periods of comparison include: 2010–2039 (A), 2040–2069 (B) and 2070–2099 (C), under the SRES A2 (mid-high) emissions scenario. The GFDL CM2.1 projections (outlined) fall in the mid-range of all models - half of the models show warmer temperatures and half show cooler.
(3.39 MB TIF)

Figure S8 Comparison of annual precipitation from 15 AOGCMs. Periods of comparison include: 2010–2039 (A), 2040–2069 (B) and 2070–2099 (C), under the SRES A2 (mid-high) emissions scenario. The GFDL CM2.1 projections (outlined) are in the lower half of the 15 models. Although there are several models that project significantly drier conditions than GFDL CM2.1, in general by end-of-century its projections show smaller precipitation increases (across northern Europe, along the west coasts of the Americas, and in mid-Africa) than the majority of models.
(3.87 MB TIF)

Figure S9 Comparison of mean temperature of the wettest month from 15 AOGCMs. Periods of comparison include: 2010–2039 (A), 2040–2069 (B) and 2070–2099 (C) under the SRES A2 (mid-high) emissions scenario. The GFDL CM2.1 projections (outlined) are relatively conservative - by the end of the century, projections are in the lower third of the 15 models.
(3.46 MB TIF)

Text S1 A comparison between ATSR fire data from 1996 to 2006 and MODIS Collection 5 active fire data from 2000 to 2006.
(0.02 MB DOC)

Text S2 A comparison between a decade of ATSR fire data and fires recorded in the Canadian Large Fire Database.
(0.02 MB DOC)

Text S3 Relationships estimated between historical fire occurrence and climate variables using observed (WorldClim) and simulated (GFDL CM2.1) data.
(0.02 MB DOC)

Text S4 Assessment to determine if ignition might limit patterns of fire occurrence, based on the Human Footprint and lightning data.
(0.02 MB DOC)
