# Supplementary figures and images for: Global Pyrogeography: the Current and Future Distribution of Wildfire
Source: PLoS One. 2009 Apr 8;4(4):e5102. doi: 10.1371/journal.pone.0005102 (PMC2662419; doi:10.1371/journal.pone.0005102)

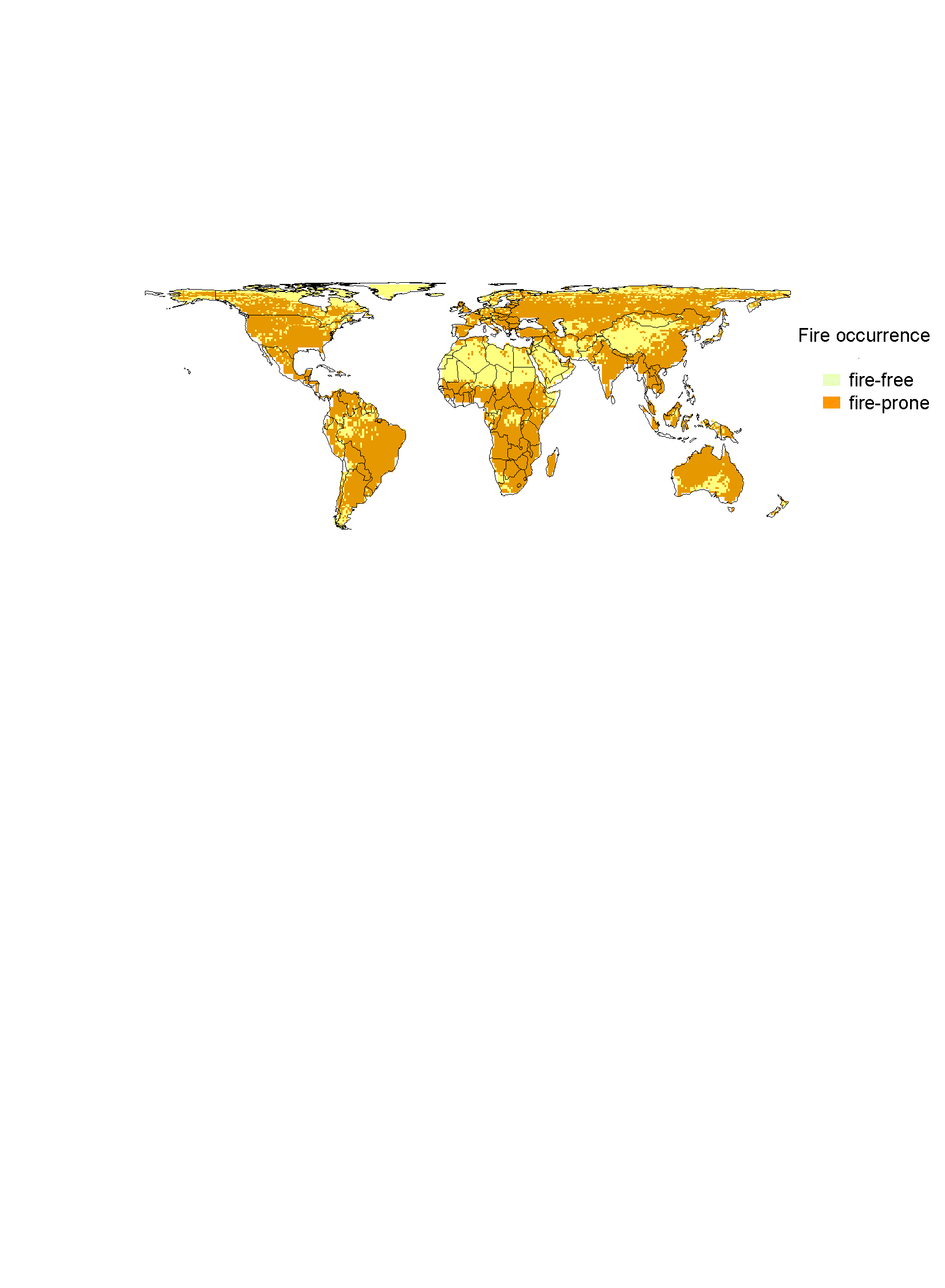

Supplement: Figure S1 — The distribution of fire detected by MODIS. Data are displayed as the occurrence of fire at a spatial resolution of 100 km, between November 2000 and December 2006. Note that areas of white within terrestrial boundaries were clipped to match the fire-climate analyses. (0.40 MB TIF) [file pone.0005102.s001.tif]

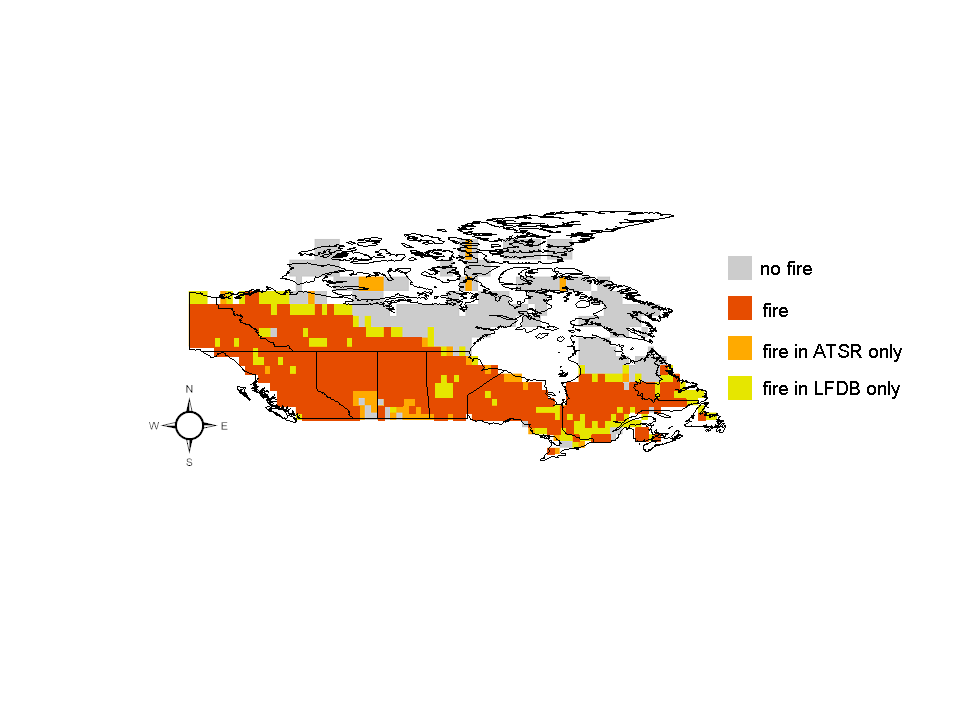

Supplement: Figure S2 — Spatial comparison between a decade of ATSR fire data and fires recorded in the Canadian Large Fire Database (LFDB). Grey represents areas where no fire was detected, red shows areas where fire was detected in both the ATSR and LFDB, orange shows areas where fires were only detected by ATSR, and yellow shows areas where fires were only documented in the LFDB. (0.09 MB TIF) [file pone.0005102.s002.tif]

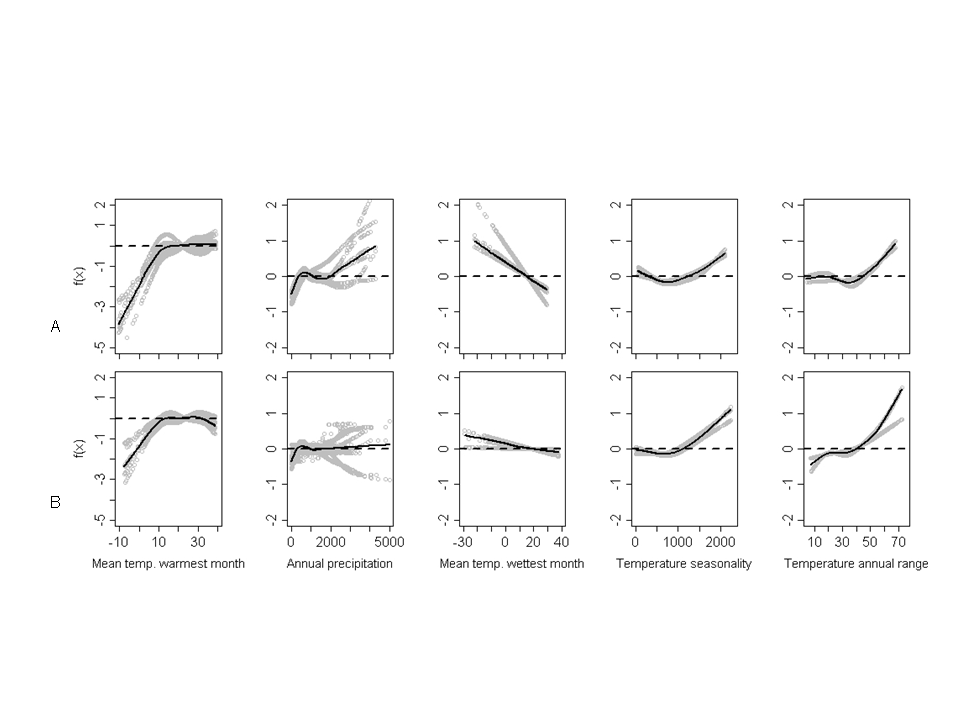

Supplement: Figure S3 — The modeled response, f(x), for the five most highly ranked climate variables of the FIRENPP ensemble. Response curves were estimated from fire occurrence and simulated GFDL CM2.1 data (A), and observed WorldClim data (B). Grey lines are estimates from each of the sub-models in the ensemble and black lines are the mean of these estimates. Descriptions of climate variables are found in Table 1 of the main text. Note that plotting axes vary among the variables; the x-axis for “Annual precipitation” is presented on a log10 scale. (0.13 MB TIF) [file pone.0005102.s003.tif]

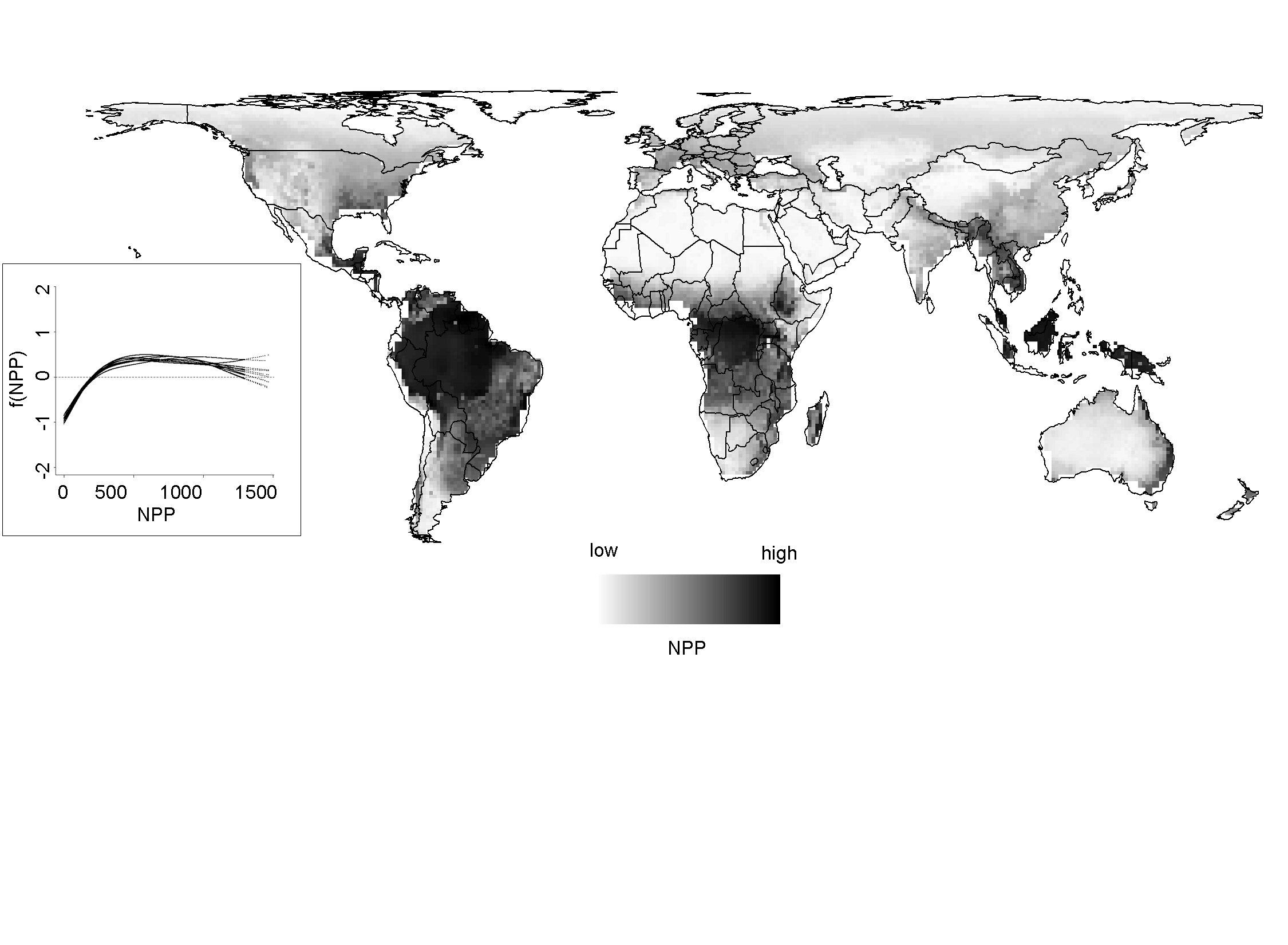

Supplement: Figure S4 — The global distribution of NPP, and the relationship between fire occurrence and NPP estimated with the ten FIRENPP sub-models. Values on x-axis are presented as approximate g C/m2/year, by dividing data (g C/0.25 decimal degree cell) by 7.7×10^8^. The values for NPP are clipped to the extent of the GFDL CM2.1 climate data used in the regression models, such that areas of white along some coast-lines indicate areas not included in the study. (1.09 MB TIF) [file pone.0005102.s004.tif]

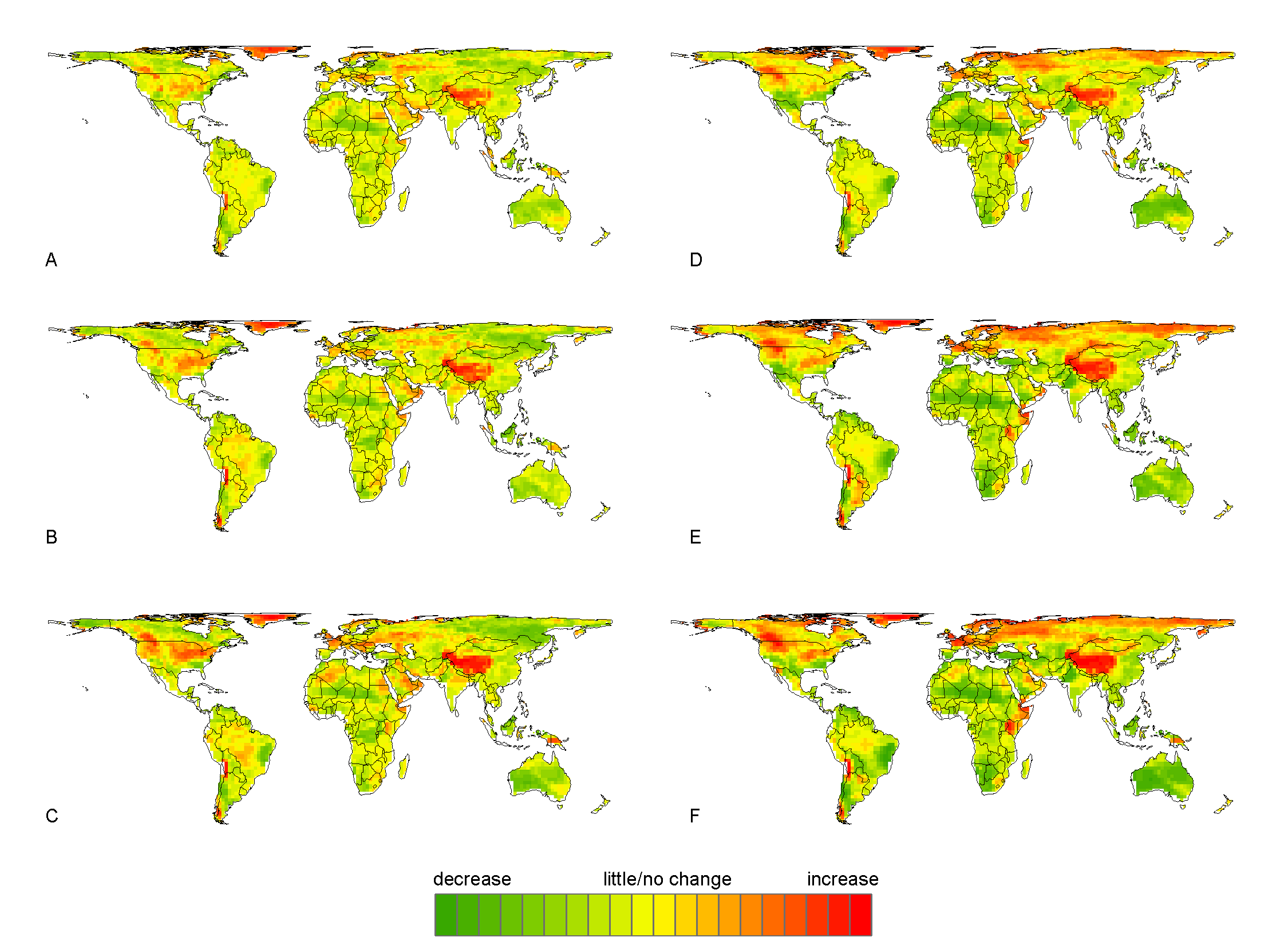

Supplement: Figure S5 — Changes in the global distribution of fire-prone pixels under the B1 (low) emissions scenario. An increase from current conditions (red) is indicated by PΔ greater than unity, little or no change (yellow) is indicated by PΔ around unity, and a decrease (green) is indicated by PΔ less than unity. Panels show the mean PΔ for the ensemble of ten FIRENPP (A–C) and FIREnoNPP (D–F) sub-models. Climate projections include 2010–2039 (A, D), 2040–2069 (B, E) and 2070–2099 (C, F). (1.38 MB TIF) [file pone.0005102.s005.tif]

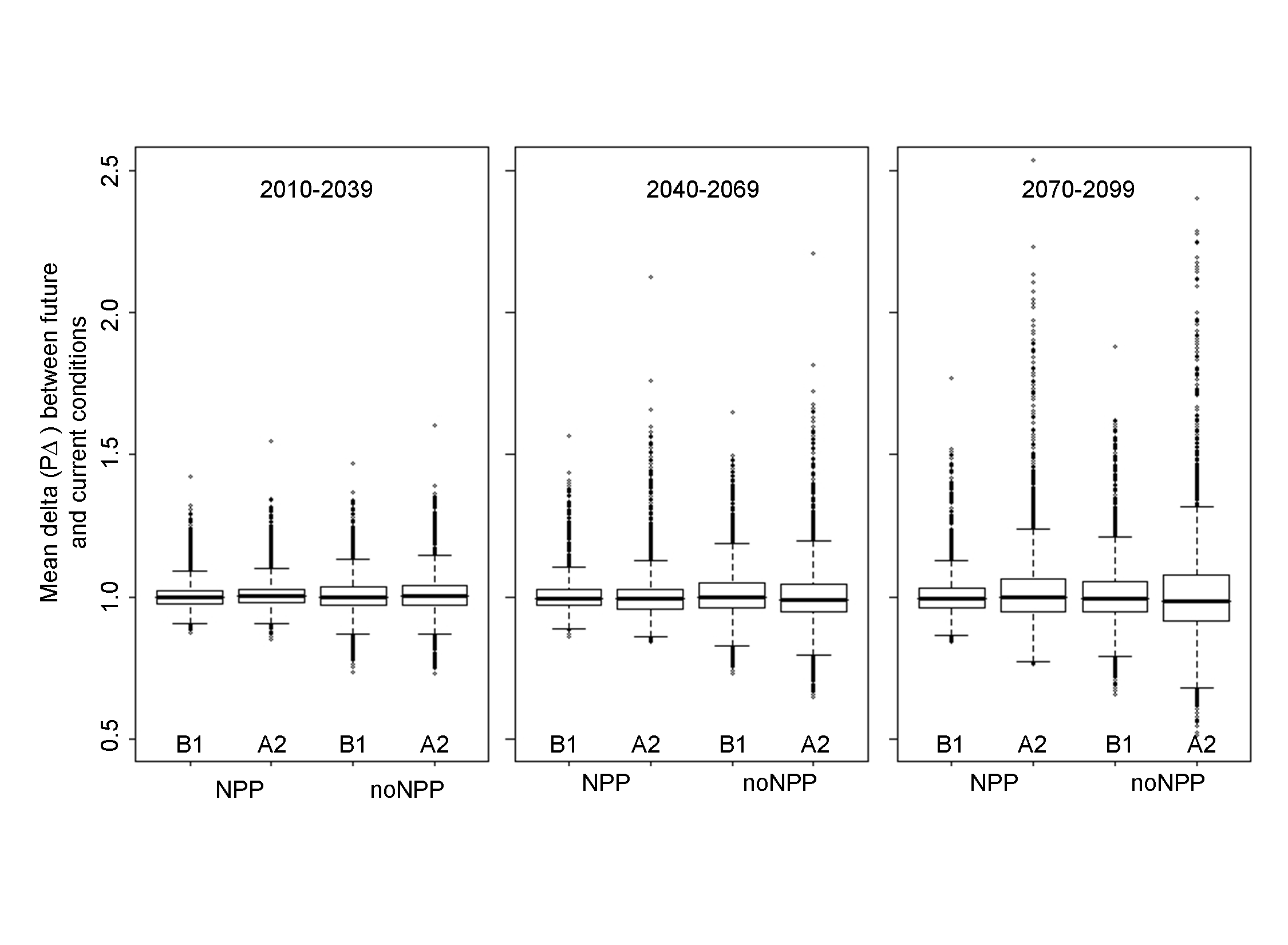

Supplement: Figure S6 — Distribution in values of change in the relative probability of fire (PΔ) under future conditions. (0.55 MB TIF) [file pone.0005102.s006.tif]

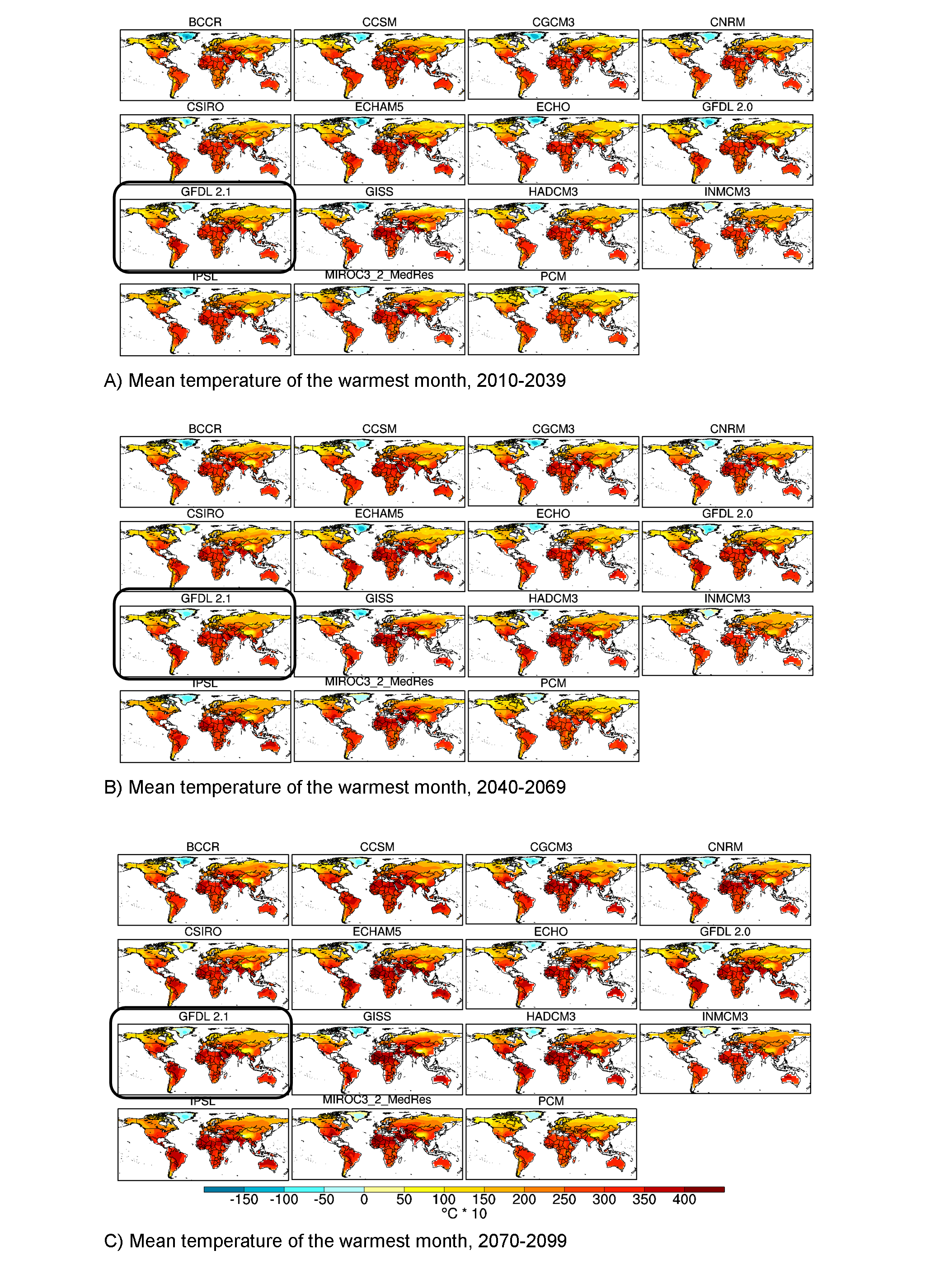

Supplement: Figure S7 — A comparison of mean temperature of the warmest month from 15 AOGCMs. Periods of comparison include: 2010–2039 (A), 2040–2069 (B) and 2070–2099 (C), under the SRES A2 (mid-high) emissions scenario. The GFDL CM2.1 projections (outlined) fall in the mid-range of all models - half of the models show warmer temperatures and half show cooler. (3.39 MB TIF) [file pone.0005102.s007.tif]

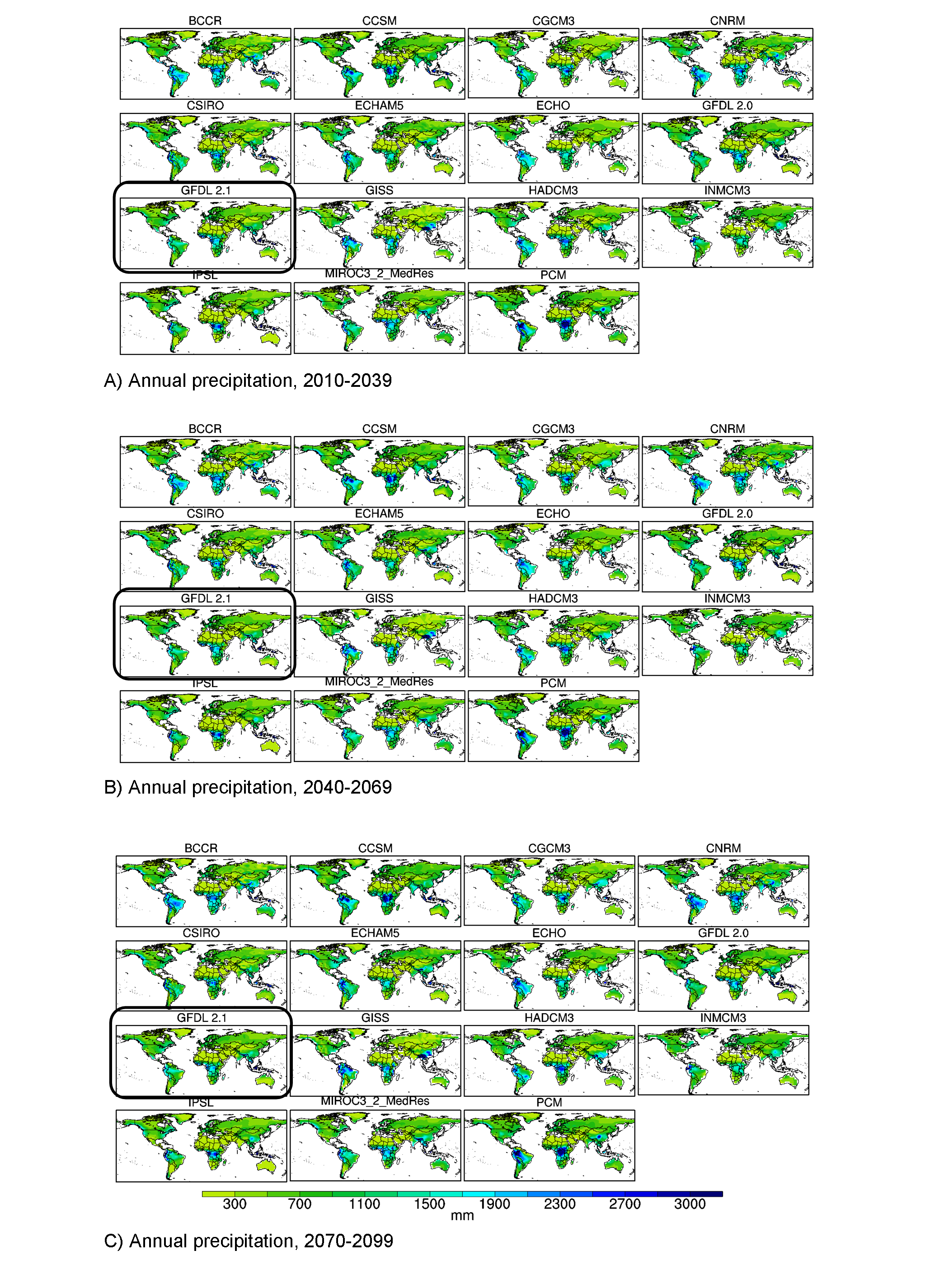

Supplement: Figure S8 — Comparison of annual precipitation from 15 AOGCMs. Periods of comparison include: 2010–2039 (A), 2040–2069 (B) and 2070–2099 (C), under the SRES A2 (mid-high) emissions scenario. The GFDL CM2.1 projections (outlined) are in the lower half of the 15 models. Although there are several models that project significantly drier conditions than GFDL CM2.1, in general by end-of-century its projections show smaller precipitation increases (across northern Europe, along the west coasts of the Americas, and in mid-Africa) than the majority of models. (3.87 MB TIF) [file pone.0005102.s008.tif]

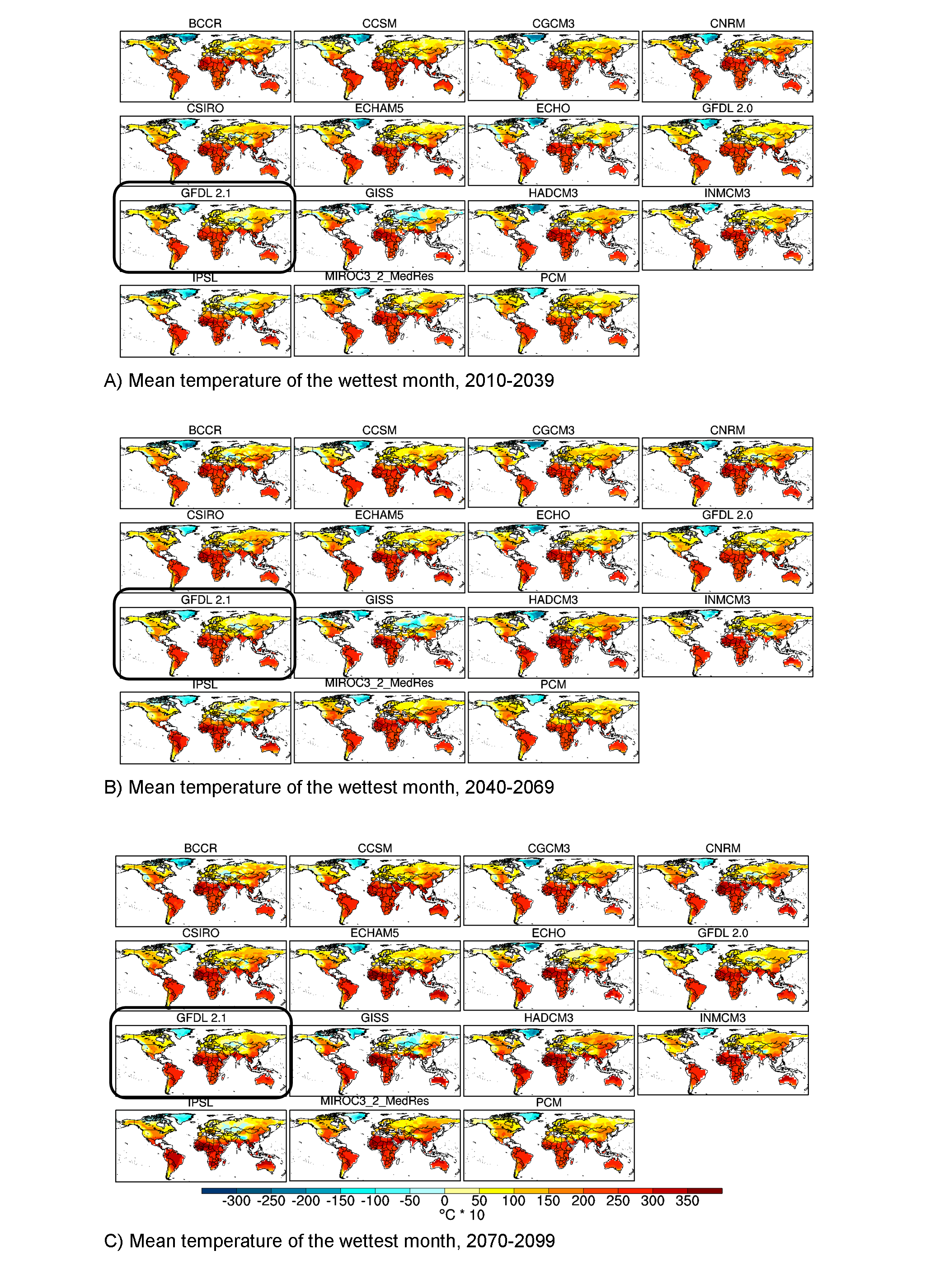

Supplement: Figure S9 — Comparison of mean temperature of the wettest month from 15 AOGCMs. Periods of comparison include: 2010–2039 (A), 2040–2069 (B) and 2070–2099 (C) under the SRES A2 (mid-high) emissions scenario. The GFDL CM2.1 projections (outlined) are relatively conservative - by the end of the century, projections are in the lower third of the 15 models. (3.46 MB TIF) [file pone.0005102.s009.tif]
