# Supplementary material for: Global Pyrogeography: the Current and Future Distribution of Wildfire
Source: PLoS One. 2009 Apr 8;4(4):e5102. doi: 10.1371/journal.pone.0005102 (PMC2662419; doi:10.1371/journal.pone.0005102)
Supplement: Text S1 — A comparison between ATSR fire data from 1996 to 2006 and MODIS Collection 5 active fire data from 2000 to 2006. (0.02 MB DOC) [file pone.0005102.s010.doc]

**Text S1. A comparison between ATSR fire data from 1996 to 2006 and MODIS Collection 5 active fire data from 2000 to 2006.**

Our primary goal in using remotely sensed data was to differentiate between fire-prone and fire-free regions of the world at a relatively coarse spatial scale. To determine whether the ATSR fire data used in this study were representative of the global fire patterns produced by other remotely sensed products we compared the ATSR-derived occurrence of fire to the occurrence pattern from the Moderate Resolution Imaging Spectroradiometer (MODIS) [36] Collection 5 active fire data detected by the Terra satellite from November 2000 to December 2006, provided as 0.5 degree Climate Model Grids (CMG). These data quantified spatiotemporal variability in fire based on detections from twice-daily satellite passes over all parts of the globe and are screened for errors and non-wildfire heat signals.

There are known differences between remotely sensed fire products [35], although these differences should be minimized at the coarse spatio-temporal resolution used in our study. Figure S1 shows the global distribution of fire generated from the MODIS data, and a simple visual comparison with ATSR data shown in Figure 1 B illustrates the two remote sensing products provide extremely consistent fire occurrence patterns and supports our proposition that results from our analyses are not contingent on the use of the ATSR data, even at northern latitudes.
