# Supplementary material for: Global Pyrogeography: the Current and Future Distribution of Wildfire
Source: PLoS One. 2009 Apr 8;4(4):e5102. doi: 10.1371/journal.pone.0005102 (PMC2662419; doi:10.1371/journal.pone.0005102)
Supplement: Text S2 — A comparison between a decade of ATSR fire data and fires recorded in the Canadian Large Fire Database. (0.02 MB DOC) [file pone.0005102.s011.doc]

**Text S2. A comparison between a decade of ATSR fire data and fires recorded in the Canadian Large Fire Database.**

An overlay of the ATSR fire data documenting the distribution of vegetation fires between 1996 and 2006 and the fires ≥ 200 ha recorded in the Canadian Large Fire Database (LFDB) between 1959 and 2002 [37] reveals a large degree of overlap between the two data sets (fire and no fire; Figure S2). This overlay supports the use of the short-term ATSR data as sufficiently representative of longer-term fire patterns at a coarse spatial and temporal resolution. In some areas, the ATSR omitted fire-prone pixels (fire in LFDB only) and, conversely, the sensor detected fires in areas where no fires have been reported in the LFDB (fire in ATSR only). Some of the latter events are likely small fires that were not recorded in the Canadian database due to its focus on large fires. In addition, though the overlay illustrates that ATSR data set omits some fires at northern latitudes, it suggests the omission is not systematic and should not substantially affect the results from our analyses. Lastly, the analysis indicated that pixels where no fire was detected by the ATSR were not strictly fire-free over the longer term, highlighting the appropriateness of the used-versus-available sampling design we used in our statistical analyses.
