# Supplementary material for: Global Pyrogeography: the Current and Future Distribution of Wildfire
Source: PLoS One. 2009 Apr 8;4(4):e5102. doi: 10.1371/journal.pone.0005102 (PMC2662419; doi:10.1371/journal.pone.0005102)
Supplement: Text S3 — Relationships estimated between historical fire occurrence and climate variables using observed (WorldClim) and simulated (GFDL CM2.1) data. (0.02 MB DOC) [file pone.0005102.s012.doc]

**Text S3. Relationships estimated between historical fire occurrence and climate variables using observed (WorldClim) and simulated (GFDL CM2.1) data.**

Modeled historical baseline climate conditions, such as the GFDL CM2.1 simulations can be useful for representing natural climate variability, but their appropriateness largely depends on the goal of the study, the variables considered, and spatiotemporal scale of interest [51]. In this case, the goal of our study was to identify broad gradients in fire-environment relationships and then use climate change simulations to project potential changes in the future distribution of fire. To that end, we first evaluated the agreement between data provided by GFDL CM2.1 simulated climate outputs for 1961–1990 [31] and the WorldClim observed data [30] for the historical reference period 1950-2000. We did this by comparing estimated regression coefficients from the sub-model ensembles (see *Regression modeling* in the main text) for the FIRE_NPP_ and FIRE_noNPP_ scenarios from both climate datasets. Overall, we found that the estimated relationships were very similar between simulated (A) and observed (B) data (Figure S3). In particular, differences between the two top variables selected in a majority of the statistical sub-models, mean temperature of the warmest month and annual precipitation, were minimal. Variation between datasets in the estimates for annual precipitation occurred at the upper tail of the distribution where there was limited information in either model set, as shown by variation among sub-models in the ensemble. However, relatively few areas globally experience these high levels of precipitation: only 1.2 % (A) and 1.3 % (B) of sample points were greater than 3 000mm (3.5 on log_10_ scale in Figure S3). These results strongly suggest that that for the purposes of this study, historical simulations from the GFDL CM2.1 can be used directly in our pyrogeography. Importantly, the statistical models built on these simulations can then be seamlessly used with future climate simulations to generate projections of fire.
