# Supplementary material for: Global Pyrogeography: the Current and Future Distribution of Wildfire
Source: PLoS One. 2009 Apr 8;4(4):e5102. doi: 10.1371/journal.pone.0005102 (PMC2662419; doi:10.1371/journal.pone.0005102)
Supplement: Text S4 — Assessment to determine if ignition might limit patterns of fire occurrence, based on the Human Footprint and lightning data. (0.02 MB DOC) [file pone.0005102.s013.doc]

**Text S4. Assessment to determine if ignition might limit patterns of fire occurrence, based on the Human Footprint (HF) and lightning data.**

One assumption inherent in this modeling effort was that ignitions would not be scarce enough in a 100-km grid cell to keep fire from occurring in an otherwise suitable environment. We tested the assumption that ignitions were not limiting to the observed distribution of fire at our 100-km study resolution by cross-tabulating values of human activity as measured by the human footprint (HF [33]), lightning flashes [34], and NPP [32].

The HF dataset was created at a 1 km resolution from nine data layers describing human population pressure (population density, population settlements), human land use and infrastructure (built up areas, night time lights, land use/land cover), and human access (coastlines, roads, railroads, navigable rivers). The index is normalized by biome and realm. We aggregated the HF to a 100 km resolution by taking the mean value of all constituent pixels. There are documented caveats about the HF: it is a coarse resolution, and has errors of commission and omission. However, it gives a global overview of human distribution arguably adequate for our study design.

The efficiency of individual lightning strikes in igniting a fire is affected by variation in lightning properties such as polarity, multiplicity, or current; moisture properties of forest fuel resulting from recent weather conditions; and rates of combustion that vary with fuel type. Although these elements can not be explicitly quantified using data available at a global extent, the occurrence of lightning provides a simplified metric quantifying the opportunity for lightning fire. We quantified the distribution of lightning activity around the world with the NASA Global Hydrology and Climate Centre Lightning Team’s 0.5 degree high resolution annual lightning climatology [34]. The data provide lightning estimates of flashes/km^2^/year collected from LIS and OTD satellite sensors between 1995 and 2005.

Because vegetation is necessary for vegetation fires, we screened out locations where NPP was low, suggesting low amounts of biomass to burn. We defined low NPP at a value of 1 × 10^11^ gC/0.25˚/year, selected by randomly sampling NPP values from the Sahara, Taklamakan and Gobi deserts. We found there were only 39 pixels that experienced no HF, no lightning, and had NPP available to burn. These occurred at high northern and southerly latitudes and in a small patch of the Sahara desert, indicating that there are few parts of the world where ignition might be limiting at the coarse resolution of our analysis.
